# Environmental Health in Perinatal and Early Childhood: Awareness, Representation, Knowledge and Practice of Southern France Perinatal Health Professionals

**DOI:** 10.3390/ijerph15102259

**Published:** 2018-10-15

**Authors:** Claire Sunyach, Barbara Antonelli, Sophie Tardieu, Michele Marcot, Jeanne Perrin, Florence Bretelle

**Affiliations:** 1 Aix Marseille, Avignon Université, CNRS, IRD, IMBE, 13284 Marseille, France; jeanne.perrin@univ-amu.fr; 2 Pôle Femmes-Parents-Enfants, Centre Pluridisciplinaire de Diagnostic Prénatal, AP-HM, 13915 Marseille, France; 3 Réseau Méditerranée, Réseau de Périnatalité PACA, Corse, Monaco, 13015 Marseille, France; barbara.antonelli@hotmail.fr (B.A.); michele.marcot@reseauperinatmed.fr (M.M.); 4 Santé Publique, Évaluation Médicale, AP-HM, Aix Marseille Université, 13284 Marseille, France; sophie.tardieu@ap-hm.fr; 5 Pôle Femmes-Parents-Enfants, Centre Clinico-Biologique d’Assistance Médicale à la Procréation, AP-HM, 13915 Marseille, France; 6 Pôle Femmes-Parents-Enfants, Service Gynécologie-Obstétrique, AP-HM, Hôpital Nord, 13915 Marseille, France; florence.bretelle@ap-hm.fr; 7 Aix-Marseille-Univ, IRD, AP-HM, MEPHI, Marseille Infection, 13284 Marseille, France

**Keywords:** environmental toxicants, reproductive health, environmental health, perinatal health professionals, attitudes, perception, practices, preventive attitude

## Abstract

The exposure of adults of reproductive age as well as pregnant women and children to environmental contaminants is of particular concern, as it can impact fertility, in utero development, pregnancy outcomes and child health. Consequently, the World Health Organisation (WHO) and international societies advocate including Environmental Health (EH) in perinatal care, yet perinatal health professionals (HPs) hardly put these recommendations into practice. In 2017, a cross-sectional study was performed in a large panel of perinatal HPs in south-eastern France with the aim of painting a picture of their current attitudes, representation, knowledge, and training expectations. Quantitative and qualitative information was collected via auto-questionnaire. Questionnaires were completed by 962 participants, mainly midwives (41.1%), physicians (25.6%) and nursery nurses (11%). Indoor/outdoor air quality and endocrine disruptors were the best-mastered topics, whereas electromagnetic fields and diet gave rise to unsure responses. Overall, perinatal HPs were ill-trained and -informed about the reproductive risks linked to daily environmental exposure. HPs reported scarce knowledge, fear of patient reaction and lack of solutions as the main barriers to providing information regarding EH to the public. Our findings highlight the need to set up EH training programmes focused on scientific knowledge and to provide simple messages and tips to help perinatal HPs deliver advice to populations to mitigate exposure to environmental toxicants.

## 1. Introduction

The general population is widely exposed to hundreds of different environmental contaminants: e.g., solvents, pesticides, phthalates, and atmospheric pollutants, some of which are endocrine-disrupting chemicals. Accumulating evidence regarding human health issues linked to ubiquitous and life-long exposure to chemicals is currently a subject of marked concern [1]. Pregnant women, developing foetuses and toddlers are particularly vulnerable [2]. The environment, before conception and during pregnancy and infancy, is linked with adverse reproductive outcomes and pregnancy complications: miscarriage, pre-eclampsia, preterm birth, low birth weight, congenital defects, infertility, risk of later non-communicable diseases in adulthood (NCDs), and possibly transgenerational threats [3,4,5]. These notions are encapsulated in the Developmental Origins of Health and Disease (DOHaD) hypothesis [6,7].

The European Parliament Regulation (EC) No. 1272/2008 recognizes that non-hereditary adverse effects in offspring, or interference with reproductive functions, including sexuality and fertility, can occur by in utero exposure to reprotoxic agents through inhalation, ingestion, and skin penetration. Likewise, the American Society for Reproductive Medicine and other international learned societies have issued formal statements that the environment can adversely affect human reproductive health [2,8,9,10,11], making clear that Environmental Health (EH) should be included in perinatal HP daily practice.

Many reprotoxic chemicals are hidden in commonly used personal care products, linings of food containers, textile treatments, and foods, as well as in cleaning, do-it-yourself (DIY) and gardening products. Parents, particularly mothers, are gatekeepers of the developing embryo, foetus and young child’s future health as well as intergenerational impact of environmental toxicant on health. Couples should avoid or mitigate exposure during pregnancy, if not before conception and during early childhood. Perinatal HPs are naturally pivotal to this environmental health education and protection, as they can provide information, education and counselling regarding reprotoxic environmental toxicants [1,12]. However, there is a large body of literature and information that is not always compelling. As a result, pregnant women are poorly informed about reprotoxic exposure during pregnancy [12,13,14]. Daily medical practice and new prevention policies need to incorporate this emerging knowledge, thereby connecting early-life exposures and later-life disease. This process largely relies on the health professional’s position and familiarity regarding issues of environmental exposure in the field of perinatology.

Grason and Misra [15] in 2009 reported the scarcity of data regarding knowledge and attitudes among perinatal HPs and about their perception of the risks associated with pregnant women’s exposure to environmental contaminants. Far from all perinatal HPs are knowledgeable regarding EH. In the United States, one out of 15 obstetricians [12] and one out of five paediatricians [16] reported having been informed on this topic. In France, 24% of general practitioners were trained [17]. In the United States, less than 20% of obstetricians acknowledged routinely inquiring about environmental history exposure [12], while in France, 50% declared doing so. Interestingly, a recent study investigated whether perinatal HPs in Auvergne, central France, were aware of the risks incurred by environmental exposure during pregnancy and of the need to give preventive advice to pregnant women [18]. To get better insight about current attitudes, representation, knowledge and awareness of perinatal health professionals regarding environmental health in France, we conducted a survey in Provence-Alpes-Cotes d’Azur (PACA) south-eastern France. PACA is the third most important French region in terms of the number of births, with 57,689 births in 2016 [19]. It accounts for 7.3% of the total number of birth (785,000) in the country. It is also the third region in term of perinatal HP demography.

## 2. Materials and Methods

With the aim to explore the attitudes, knowledge and expectations of perinatal health professionals, we conducted a prospective observational study using a self-administered questionnaire. The method involved a “triangulation” strategy in which both qualitative and quantifiable data were collected and analysed to address both subjective and objective aspects of the problem of environmental health. The secondary objective of the study was to be able to propose training schemes adapted to professional needs and wishes. No nominative data were entered in the data base; our study was, therefore, not subjected to institutional approval in accordance with French human research law.

### 2.1. Study Population

The study population consisted of perinatal health professionals practising in hospitals as licensed professionals in Mother and Infant Care Centres, structuring day care for young children of the Provence Alpes Cote-d’Azur region, who answered the questionnaire. The PACA region comprises six districts (Bouches du Rhone, Alpes Maritimes, Var, Vaucluse, Hautes Alpes, and Alpes de Haute-Provence). Monaco and Corse were also included in the study, as they are included in the perinatal HP network “Réseau Mediterranée”. The study population was mainly made up of gynaecologists-obstetricians (GOs), general practitioners (GPs), paediatricians (Ps), midwives (MWs), and nurses (Ns). Other professionals were also represented.

### 2.2. Study Design

Two identical versions of the questionnaire were disseminated. One online version was sent to HPs, using the directory of the perinatal health professional network “Réseau Mediterranée” PACA-CORSE-MONACO, which includes up to 5000 professionals’ e-mail addresses. A printed version was distributed during scientific meetings or provided to health establishments upon request.

Data were collected between January and April 2017 by a standardized anonymous self-administered questionnaire. The questionnaire was structured with five parts. To first explore perinatal HPs’ representations, we chose the free association method using “environmental health” as an inductive expression. HPs were asked to give three words or expressions. Note that they were informed of the WHO EH definition to avoid any confusion with the semantic of ecology. A second part was a Likert-based questionnaire. Participants were asked to rate the extent to which they agreed or disagreed with 25 true or false assertions. The response scale used the following anchors: 1 = fully disagree, 2 = partially disagree, 3 = does not know, 4 = rather agree, and 5 = strongly agree. General knowledge regarding the 11 following items was investigated: environmental toxicant mechanism of action, general effects, endocrine disrupting chemicals, indoor/outdoor air quality, food/diet, cosmetics, electromagnetic fields, early childhood, nursery and childcare products, and impacts on fertility and lead exposure. These items were randomly organized. When necessary, i.e., when the assertion was wrong and when the proposed response was inverted, the score was inverted. Consequently, knowledge was considered adequate and good when the score was close to 5. When the score was ≤3 the topic was considered poorly mastered. Responses were correct when in accordance with the literature or were wrong. Responses were extreme (1 “fully disagree” or 5 “strongly agree” on the Likert-based scale i.e., the responder was sure) or hesitant (2 “partially disagree” or 4 rather agree” on the scale). True and false assertions were randomly organised. We included two additional multiple choices questions. Firstly, to better understand what perinatal HPs knew about lead exposure, we proposed five potential sources of lead exposure; secondly, four potential sources of EDs exposure were proposed. A third part proposed six Likert-based questions to evaluate HPs’ concerns regarding EH in the context of their routine practice (response scale was 1 = not, 2 = not really, 3 = mildly, 4 = rather, and 5 = fully concerned), if they felt the need to inform their patients, that it was difficult to do, or to address their patients to other HPs experts in EH (response scale was 1 = never, 2 = seldom, 3 = sometime, 4 = often, and 5 = always). The participants were requested to scale their knowledge (1 = null, 2 = poor, 3 = average, 4 = good, 5 = excellent), and their interest in training concerning these issues (response scale was 1 = not, 2 = not really, 3 = mildly, 4 = rather, and 5 = fully interested). One open-ended question asked what the participants felt were the main environmental problems in the geographic location of their professional practice. One inquired about the reasons they thought they faced difficulties to inform their patients. The last part concerned general socio-demographic and professional characteristics (age, gender, professional postcode, year of qualification, and initial or secondary training in environmental health). At the end of the questionnaire, it was proposed to the participants to volunteer to become “an active professional” in environmental health, i.e., become an EH expert, to pass on information to their peers or to the public in their area. Some questionnaires were returned incomplete for general information (age, date of graduation, and length of practice) and they were, nevertheless, analysed for the environmental portion. The questionnaire is presented in the Supplementary Materials (Table S1).

### 2.3. Thematic Content Analysis

To analyse the content of the free words or expression associated to environmental health, we used the content analysis approach as originally described by Bardin [20]. Theme and sub-categories were determined after familiarisation with the data. Qualitative data from the questionnaires were analysed, using a thematic analysis chart that was designed to identify the themes cited and to make comparisons to highlight recurrent issues and differences. Frequency of words and expressions occurrences were determined using Excel.

### 2.4. Statistical Analysis

Quantitative data were extracted from the questionnaires and analysed using Excel (Microsoft, Redmond, WA, USA) and SPSS software (IBM Analytics, Chicago, IL, USA). Descriptive and variance analysis were performed to compare the responses of each group. The internal consistency of the data was tested using Cronbach’s alpha coefficient, which was considered satisfactory from 0.7 [21].

To allow variance and Chi-square test analysis, we formed three groups of occupation (population size > 30): midwives (G1), physicians (G2) and non-medical professions (G3). Regarding the area of practice, two Alpine areas (Alpes de Haute-Provence and Hautes-Alpes) were combined. Corsica and Monaco were not considered, as the figures were too small. Finally, establishments of health, licensed professionals, Mother and Infant Protection Centres and nurseries were the modes of professional practice considered. For intergroup comparison of the “knowledge” variable, Chi-square analysis were performed to allow an analysis of category. ANOVA exact Fisher test was performed to compare scores.

## 3. Results

### 3.1. Response Rate and Study Sample Characteristics

**Figure 1:**
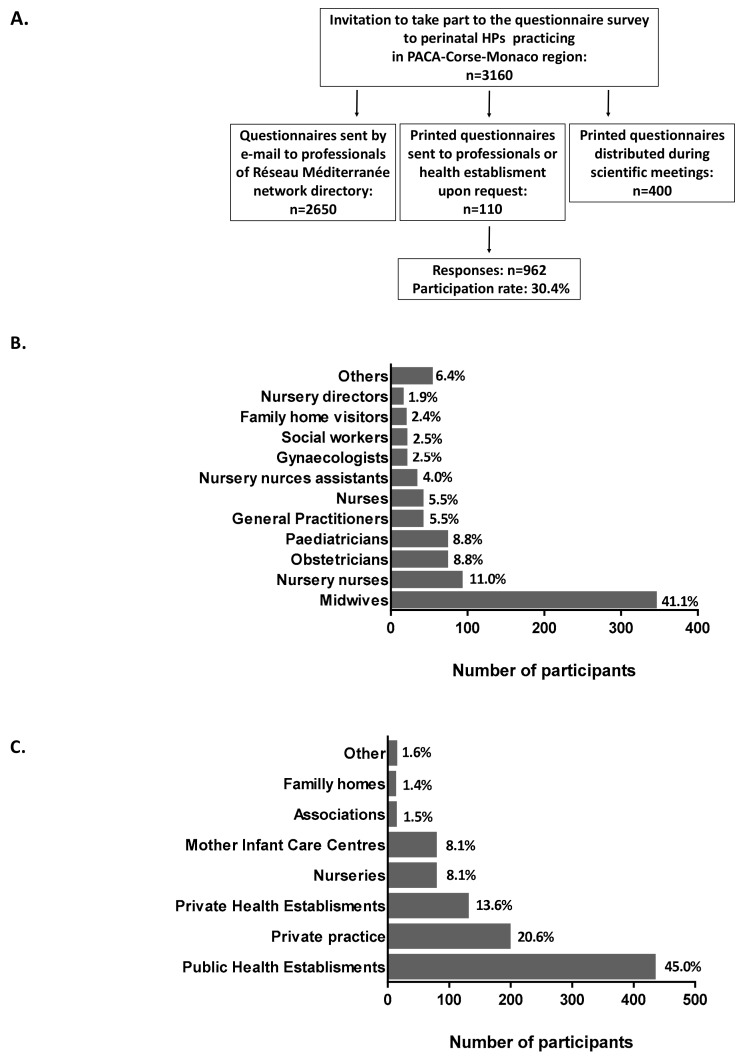
Participation of perinatal HPs to the study. (**A**) Flow chart of auto-questionnaire HPs recruitment. (**B**) Number of participants by profession involved in perinatal care. (**C**) Number of participants by model of practice.

A total of 962 completed questionnaires were collected and analysed. The questionnaire survey participation rate was 30.4% (Figure 1A). Twenty-eight professions were represented. Frequencies of main professions of participant who replied to the invitation to complete the questionnaire, are presented in Figure 1B. A complete list of professions who relied to the survey and effectives is provided in the Supplementary Materials (Table S2). Midwives had a large response rate (41.1%), and physicians (obstetricians, gynaecologists, paediatricians and general practitioners (GP)) accounted for 25.6% of the sample. The majority of HPs (45.0%) practised in public health establishments (Figure 1C) and 20.6% in private practice. Socio-demographic characteristics and geographic area of practice of the questionnaire samples are detailed in Table 1.

### 3.2. Environmental Health Representations

Free association questioning gave rise to 2612 words or expressions out of which, 55 words were rejected on the grounds of ambiguity or because it was impossible to classify them reliably. After semantic clustering, 17 themes were extracted “Toxic agents and environmental health risks” accounted for 613 (68%) words or expressions occurrences (e.g., “pollution(s)”, “pollutants”, “impact of pollution”, “pesticides”, “food quality”, “Endocrine disruptors” …). It was followed by 293 (32.5%) word or expression occurrences referring to the theme 2: “collective means of action (society and communities)” (e.g., “protection”, “prevention”, “care”, “health promotion”…). A complete thematic analysis of the content, effectives, theme and subcategories are presented in Supplementary Materials (Table S3). Environmental health appeared as a risk for 69.6% of the participants, mainly for human health (68%) and ecology (1.6%). Interestingly, in some cases, the approach was positive (18.2%), with EH envisioned as the improvement of health rather than degradation. Participants referred to means of action in 40.6% of the cases, ranging from collective and social (32.5%) to individual, i.e., behavioural (8.1%), such as “earth protection and respect for the environment”, “develop prevention, inform and raise awareness of the public”, “patient care proposition”, “healthy way of life”, “physical activity”, “precautionary principle”. Barriers to protecting health from environmental exposure were cited by 15.5% of the respondents, i.e., “impact of socio-demographic factors in environmental health”, “Economical and commercial issues”, “political and economic choices”. Finally, 20.9% raised the importance of including EH in our society. Overall, a rather pessimistic representation of EH was raised, and 2.5% of perinatal HPs surveyed thought that the general population and decision-makers were aware of the problem. Medical professionals (i.e., midwives G1 and physicians G2) considered EH in terms of risks (pollutants, endocrine disruptors, and diet) and non-medical professionals (G3) reported adopting an approach oriented towards pragmatic action implementation.

### 3.3. General Knowledge in Environmental Health

#### 3.3.1. Item Analysis

Knowledge of perinatal HPs regarding EH was investigated in the second part of the questionnaire. Questions and expected responses, general mean score obtained by HPs, and the numbers and proportion of correct/extreme, correct/unsure, and wrong responses are presented in Table 2.

Out of 25 questions, 20 were correct, i.e., in agreement with the literature for more than half of the responders. The question “mothers are advised to feed fish to children under 3 at least two times a week” gave rise to 24.6% correct and 45.5% wrong responses. The question “It is generally advised to wait 2 months before placing a baby in his freshly redecorated/refurbished bedroom” raised 48.3% “does not know” responses. For the three questions “It is generally advised to wait 2 months before placing a baby in his freshly redecorated/refurbished bedroom”, “Consuming organic food can reduce obstetric complications” and “Pregnant women are advised to eat fatty fish at least two times a week”, the responses were rather dispersed between right, wrong and “does not know” answers. In addition to the accuracy indicator, certainty was analysed. Interestingly, out of 25 questions, 14 gave rise to more than 50% correct and extreme responses (Table 2). Multiple-choice questions indicated that only 10.5% of the sample identified the potential sources of lead exposure. “Some paints” was checked off in 98% of the questionnaires, but only 47% identified kohl eyeliners. Concerning sources of Endocrine Disrupters (ED), 66% identified all and only ED sources proposed.

#### 3.3.2. Analysis by Topic

**Figure 2:**
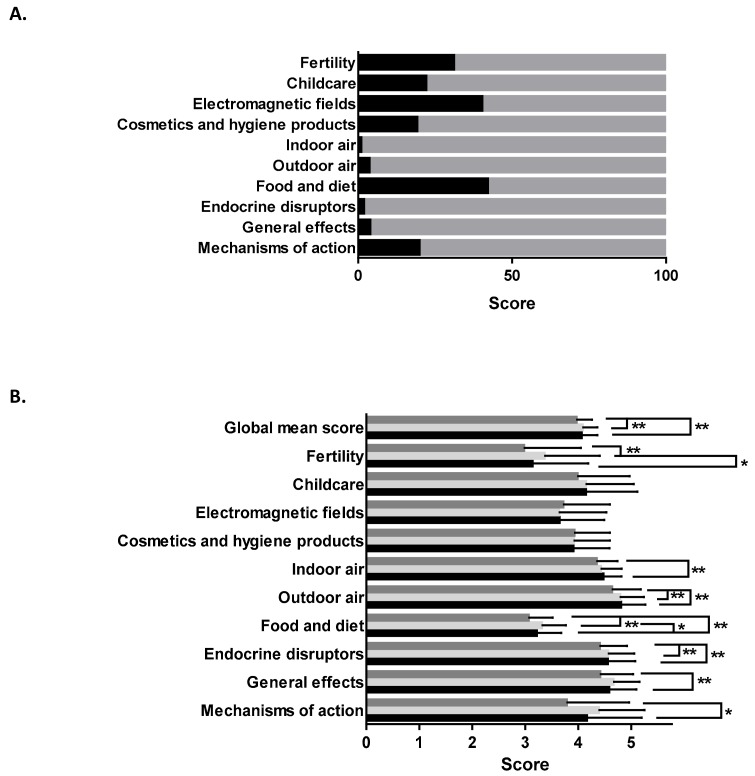
Mastery of environmental health topics. (**A**) Perinatal HPs mastery of environmental health topics. For each professional, scores were calculated using the 1 to 5 Likert based scale. Black bars: topic not mastered, score ≤ 3; grey bars: topic mastered, score > 3. (**B**) Inter group comparison of scores obtained by professional groups for each environmental topic. Black bars: (G1) midwives; light grey bars: (G2) physicians; dark grey bars: (G3) non-medical professions. * *p* 0.01 to 0.05; ** *p* 0.001 to 0.01.

All the 25 propositions presented in Table 2 were grouped by different topics (Figure 2A): environmental toxicant mechanism of action (route of exposure ), general effects (delayed effect and reversibility), endocrine disrupting chemicals (plastic food containers, baby bottles, in utero exposure, warming in plastic containers), indoor/outdoor air quality (refurbishing, smoke of cigarettes, air refreshers, incense, spray), food/diet (fish consumption, vegetable processing, organically grown fruits and vegetables), hygiene and cosmetics (rinse-off products), electromagnetic fields (baby phone), childcare, and impacts on fertility. We considered a theme mastered when the general score obtained was >3 on the 1 to 5 scale. Figure 2A shows that with more than 90% > 3 score, indoor (99.1%), outdoor air (96.4%), endocrine disruptors (98.1%) and general effects (96.2%) were the best mastered topics, whereas food and diet and electromagnetic fields were less mastered, with less than 60% of scores > 3 (58% and 59.8%, respectively).

#### 3.3.3. Intergroup Analysis According to Profession

Variance analyses were performed to unravel potential differences due to professional group (G1 midwives, G2 medical professions, and G3 non-medical professions). The results presented in Figure 2B show that the pooled global score obtained was significantly impacted by the professional group (*p* < 0.001), with better scores obtained in groups G1 and G2 (4.06 ± 0.30 and 4.08 ± 0.28, respectively) vs. G3 (3.96 ± 0.3). The same trend was observed for outdoor air and EDs. For fertility, better scores were obtained in the G2 group (3.36 ± 1.05), to G1 (3.13 ± 1.06, *p* < 0.01) and G3 (2.97 ± 1.08, *p* < 0.001). For the indoor air topic, the G1 group obtained a significantly better score (4.47 ± 0.35) than the G2 group (4.34 ± 0.40), *p* < 0.001. For questions related to food and diet, the score obtained by the G2 group was 3.31 ± 0.46 and was significantly higher than the G1 group 3.21 ± 0.47, *p* < 0.5 and significantly better itself than the G3 score (3.06 ± 0.46, *p* < 0.001). Regarding “general effect of exposure” and “mechanism of action” knowledge, the G1 group obtained significantly better scores than the G3 group (4.58 ± 0.52 vs. 4.41 ± 0.63, *p* < 0.001) and lower scores than the G2 group (4.16 ± 1.04 vs. 4.38 ± 0.87, *p* < 0.5). Overall, perinatal HPs have sound knowledge regarding environmental risks on health. The level of confidence is lower for some aspects, i.e., cosmetics, mode of action, childcare, and fertility (“correct and unsure response”, Table 2) and is illustrated by >3 general scores included in an interval ranging from more than 60% and less than 95% of the total population (Figure 2A). As a whole, non-medical professionals obtained lower scores than midwifes and physicians.

### 3.4. Environmental Health Subjective Aspects

**Figure 3:**
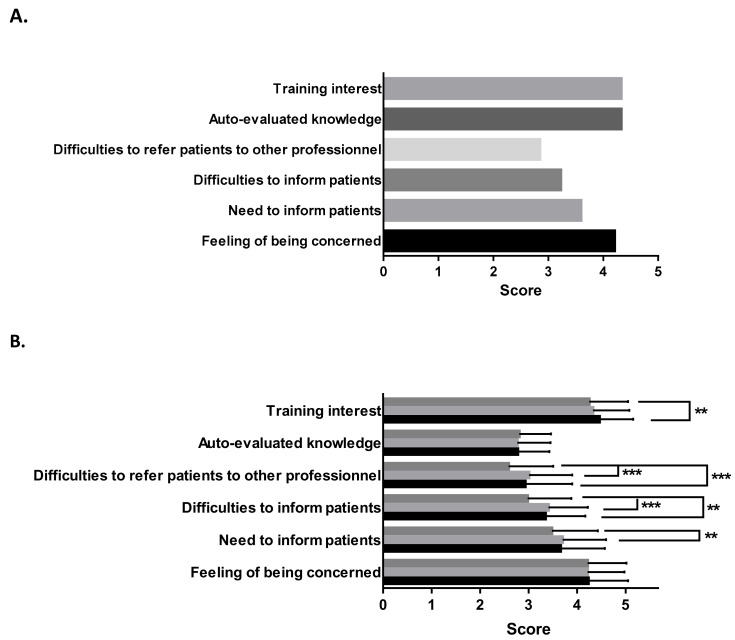
Subjective dimension formulated by professionals. (**A**) General scores obtained by Perinatal Health professionals for each subjective topic. Scores were calculated using the 1 to 5 Likert based scale. (**B**) Intergroup comparison were performed for the scores obtained for each subjective aspect by each group of profession. Black bars: (G1) midwives; light grey bars: (G2) physicians; dark grey bars: (G3) non-medical professions. ** *p* 0.01 to 0.001; *** *p* < 0.001.

We have been able to further investigate subjective aspects of perinatal Environmental Health in the perinatology field: perinatal HPs’ feeling of being concerned about EH, whether or not they felt necessary to inform patients about it, if they found it difficult to do so, and if they sometimes felt the need to refer patients to other professionals experts in EH when environmental topics were raised. Our results indicated that perinatal HPs were concerned about EH (4.21 ± 0.81, Figure 3A), being “rather concerned” and “fully concerned” for 41.5% and 41.3%, respectively, of them. Variance analysis did not show any differences either between groups of professionals (Figure 3B) or the establishment of practice (data not shown) or area of practice (data not shown). In daily practice, if perinatal HPs acknowledged the need to inform patients regarding EH (global score 3.6 ± 0.94), only 16.4% sent it must “always” be done, 40.8% “sometimes” and 31.4% “seldom”. Medical professionals (G2) were more prone to inform patients about environmental risks than members of non-medical professions (G3) (Figure 3B *p* < 0.5). In addition, professionals working in health establishments also reported the need to relay this information more than those practising in private or in nurseries (*p* > 0.001). 

Professionals declared facing difficulties to inform patients (3.23 ± 0.0.87), 44.8% “sometimes”, 32.4% “often”, and 5.4% “always”. Midwives (G1) experienced more difficulties than G2 and G3 to inform patients (*p* < 0.001). Transferring patients to colleagues and experts was seen as difficult (2.85 ± 0.96); once again, the G1 group reported facing difficulties in term of information to provide to patients more often than the G2 and G3 groups (*p* < 0.001). The mean score of auto-evaluated knowledge was 4.33 ± 0.67, with no significant differences between groups. Half (56.7%) of the participants declared having a medium level of knowledge in EH. At both ends, only 4.1% confessed no knowledge and 0.2% an excellent level. As a whole, perinatal HPs recognized the need to be trained (4.33 ± 0.79), with 40.4% being “rather” and 48.3% “fully” interested. Professionals working in Mother and Infant Care Centres declared being significantly more interested than those working in nurseries (*p* < 0.001). Note that HPs admitting a low level of knowledge are not necessarily those asking for training (*p* = 0.128). We further asked HPs to elaborate reasons why they faced difficulties in confronting EH in an open-ended question. Out of the 547 persons who responded to this question, 685 terms were collected. Qualitative thematic analysis is presented in Table 3. Briefly, three fourth (73.7%) of the sample, cited paucity of knowledge as the main reason, followed by the fear of patient reactions (9.5%) and lack of solutions to provide (11.5%).

An additional open-ended question was intended to provide insight on environmental topics that perinatal HPs felt they were facing in daily practice in their geographic area. Across 729 questionnaires, 1178 terms were recorded, falling into 13 groups after semantic analysis: pollution, daily products (personal and cleaning), inadequate housing, indoor air quality, pesticides, occupational exposure, ED, diet, toxic consumption, electromagnetic waves, waste, socio-economic level, and noise. The thematic analysis is presented in Table 4. A large number of perinatal HPs (40.1%) cited general sources of pollution, followed by pesticides (13.6%); EDs were also evoked (8.5%), particularly by HPs from the G2 group (*p* < 0.01), compared to the G3 group. Individual exposures were also cited; 10.5% of HPs were concerned about inadequate housing and 5.2% about indoor air quality, and 20.2% cited daily cosmetics and hygiene products. The G3 group raised this last topic significantly more often than the G1 group (*p* < 0.001). Occupational exposures were also mentioned, in particular, for exposures encountered in hospital establishments in relation with their own risk of exposure. Note that 362 out of 840 professionals surveyed (34.1%) agreed to become “active EH professionals”, in full agreement with fact that they felt concerned by the EH topic.

### 3.5. Training and Information

**Figure 4:**
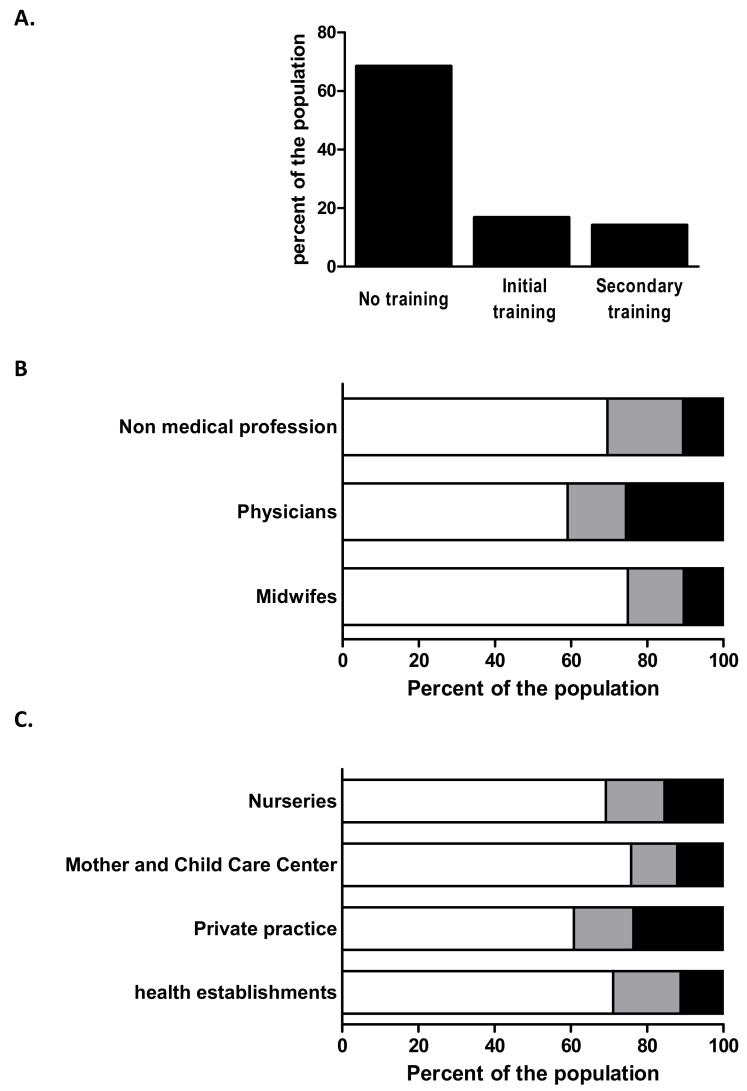
Training of perinatal health professionals in Environmental health. (**A**) Percent of the total population of perinatal health professional having followed none, initial and secondary training in EH. (**B**) Percent of perinatal HPs having followed none, initial or secondary training in EH by group of profession. Whites bars: no training, grey bars: Initial training; black bars: secondary professional training. (**C**) Percent of perinatal HPs having followed none, initial or secondary training in EH by mode of practice. Whites bars: no training, grey bars: Initial training; black bars: secondary professional training.

We investigated whether perinatal HPs had received initial professional training regarding EH, if they had followed secondary training and how they obtained information. Only 16.8% (Figure 4A) of our population had heard about EHs during the course of their initial training. Attending secondary training was more frequent in professionals working in private practice than in residents (*p* < 0.001) (Figure 4C). In addition, attendance was more frequent in the G1 group than in the G3 group (*p* < 0.001) (Figure 4B). General knowledge scores were slightly higher for professionals who had been initially trained (4.03 ± 0.28) than for those who had not (4.03 ± 0.31, *p* = 0.271). In contrast, secondary training significantly improved the score (4.11 ± 0.27 vs. 4.02 ± 0.31, *p* < 0.001). Importantly, secondary training led to an increased need to inform patients about EH (*p* < 0.001) and a greater feeling of being concerned about this matter (*p* < 0.1).

**Figure 5:**
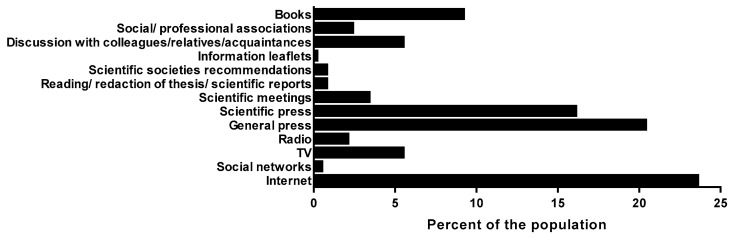
Information channels used by health professional

An important issue is the channel used by perinatal HPs to obtain information about EH. More than half of our population (61.8%) declared looking for information via the scientific press (16.1%), general press or public media (20.4%), scientific publications (16.1%) and books (9.2%). The G2 group referred to specialized media more often than the G1 and G3 groups (Figure 5).

## 4. Discussion

### 4.1. Perception and Representation of Environmental Health

Few studies have investigated the knowledge of perinatal health among care providers regarding specific exposure risks. Our study provides the first survey in south-eastern France, and a good snapshot of the current knowledge, attitudes and expectations regarding EH in a large population of professionals. The Provence Alpes-Cote d’Azur region is the third highest in France for natality rate and perinatal HP professionals’ demography. This statistic is of particular importance since PACA is also a region facing environmental challenges. Marseille is a large harbour and the city is close to a major centre of the petrol and chemical industry. The region is also at the crossroads of south-north traffic and is an agricultural centre. In addition, a rather large proportion of its population has low income and a low education level [28]. The impacts of toxicants tend to cluster in this population, thereby making it particularly vulnerable.

In the last two centuries, industrialization has prompted marked changes in our way of life, leading to daily environmental exposures. Widely used chemicals display proved or suspected health effects, which include reproductive and development issues [3,4,25]. Modern times have created new burdens and challenges for health professionals who must take into account the EH issue. Overall, the present survey indicated that in southeast France, the perinatal HPs felt concerned about EH and recognized the necessity to inform patients.

### 4.2. Attitudes and Barriers

Widespread awareness of environmental toxicants and their impact on reproductive and perinatal outcome is essential to decrease preconceptions and prenatal exposure. However, with the exception of occupational exposure, contaminated food, smoking, and alcohol and drug consumption; environmental history is rarely considered in France [17]. Consequently, when surveyed, women and couples are found to be poorly informed regarding chemical exposure prevention [13,14]. Along the same lines, Marie et al. [18] showed that HPs do not give advice regarding chemical exposure prevention, even though pregnant women requested information about environmental hazards. In our survey, among the barriers highlighted, fear of patients’ reactions was cited. These results are consistent with those of other studies [18]. This fear must be taken into account since it has recently been shown that information regarding endocrine disruptors is a concern in 68.6% of patients [14].

In 2016, Sutton and colleagues made four recommendations “to move from awareness to action” on preventing patient exposure to toxic environmental chemicals [29]. The authors particularly advocated for making environmental heath part of health care. By demonstrating the strong interest of perinatal HPs in France, the present survey and others [18] show that the time is right to set up clinical settings in which EH is fully included. However, many challenges to asking and counselling patients about toxic chemicals remain (i.e., time, communications skills and training).

### 4.3. Knowledge and Training Needs

Overall, perinatal health professionals have a sound knowledge regarding environmental health; nevertheless, some trends have emerged. The general knowledge score was inferior in the non-medical profession group. As a consequence, some special attention must be placed on the training of this group.

General topics, including indoor air and endocrine disruptors, were well mastered. The finding that almost two thirds of our population can identify ED sources is consistent with the fact that in France and Europe, these sources are largely covered by the general media and public authorities, and politicians have taken well-publicized actions. However, despite media coverage of the subject, a recent study conducted in central France showed that women do not know much about EDs [14]. It is therefore important that perinatal HPs not only answer questions asked by pregnant women but also inform about possible and simple actions that one can use to decrease exposure.

In most countries, it is generally accepted that we spend more time indoors than outdoors. Regulations and guideline values have been issued since 2010 for indoor air quality by WHO [30]. However, if HPs are conscious of indoor air as a problem, the question focusing on refurbishing the baby bedroom raised a 48.3% “does not know” response. Consequently, HPs probably poorly relay the correct message. This area is of particular concern since couples tend to refurbish their home to welcome a baby.

Endocrine disruptors are largely present in cosmetics. Rouillon et al. [14] showed that if child- bearing women consider cosmetics a potential source of ED, only 13% of them are eager to reduce their use of these products. In agreement, Marie et al. [31] recently observed that even though pregnant women are aware that cosmetics can be harmful, they continue to use them during pregnancy. Importantly, it has been shown that in a pregnant women population, the pattern of urinary phthalate metabolites and propyl paraben, among other biomarkers, varies according to the hygiene products used [32]. This finding indicates that advice can and must be given regarding cosmetic use during pregnancy, and perinatal health professionals must be ready to do so.

Among the less-mastered topics stand food and diet. Organic pollutants (PCBs and dioxins (PCDDs), furans (PCDFs) and a range of organochlorine pesticides (OCPs) have a wide range of adverse health effects, including adverse pregnancy and reproductive outcomes [33]. These exposures are largely through diet, such as frequent intake of fish, shellfish or wild foods. Scientists and HP professionals can counsel people on a number of personal lifestyle habits that can easily be changed. The skin and the fat of these foods should be removed when prepared. It is of concern to observe that the majority of our sample gave unsure responses to the questions focused on diet: “Consuming organic food can reduce obstetrical complications” and “Pregnant women are advised to eat fatty fish at least two times a week”, with responses distributed between correct, wrong and “does not know” and that “mothers are advised to feed fish to children under 3 at least two times a week” gave rise to 24.6% correct and 45.5% wrong responses, respectively. Pregnant woman are advised to eat fatty fish for intake of omega-3, however their content in heavy metal and PCB can be high. HPs should advise pregnant women to reduce their consumption of these fishes. In France the ANSES [23] has issued in 2013, clear recommendations regarding this particular point to keep in line with the benefice risk equation. It seems that 5 years later, a majority of HPs is not aware of this recommendation. As a whole dietary issues are among the topics less mastered by our perinatal HPs. This could in part be explained by the fact that some dietary (e.g., pesticides) impacts on fertility, pregnancy outcomes and development are only suspected [25]. Pesticide exposure is perceived as very high or high risk by 90.8% HPs and they receive inquiries regarding this topic [18]. Even if little is known about the potential benefice of eating organic food, it has been shown that choosing organically grown fruits and vegetable during pregnancy was associated to reduced risk of p-eclampsia [27], hypospadias and cryptorchidism [26]. It is therefore important to make patients know that preferring organic food could have a real benefice for reproduction. In addition, a recent review, showed that, peeling fruits and vegetable, can reduce pesticide residues more effectively than washing and soaking [34], it could therefore be worth transmitting this message to patients.

Magnetic waves were perceived as very high or high risk by 53.8% of a French perinatal HPs population [18]. Even if there is a lack of consensus regarding health consequences of exposure to electromagnetic waves (mobile phones, Wi-Fi), a web search indicates that consumers make inquiries. Scientific literature on the subject is poor and could explain hesitant responses to the question regarding baby phone. We believe that for this subject the “principle of precaution” should prevail.

It is well recognized that maternal and infant adverse health outcomes are linked to prenatal lead exposure. Furthermore, lead exposure’s adverse effects can be identified at lower levels of exposure than previously thought in both adults and children. Consequently, guidelines regarding the monitoring and management of pregnant and lactating women who have been exposed to lead were issued in the United States in 2010 by the Centers for Disease Control and Prevention [35], stating that “lead exposure should be kept as low as possible during pregnancy to minimize adverse outcomes”. If routine testing is not advisable, simple screening during pregnancy visits using quick questionnaires must be done, and HPs should be ready to prescribe monitoring, as recently recommended in France [36].

The need to incorporate EH in initial training is a clear conclusion of our study and has been regularly called upon by others [12,18,37]. It appeared from our survey that along with medical professions, this training must be extended to other professions in contact with families and small children, such as child care providers or social workers. Importantly, HPs asked for training done by peers accustomed to both perinatal health and EH, with emphasis on simple and pragmatic messages that are easy to deliver and easy for patients to follow. Special skills must also be acquired to convey prevention without eliciting stress in the public.

### 4.4. Limitations and Strengths of the Study

Our cross-sectional study has limitations. The study may have selection bias since the participants responded on a voluntary basis and therefore may already be involved and more aware of environmental health. The sample was large (3160 professionals were invited). The participation rate (30.4%) which was above a similar survey (11%, [18]) could have been explained by this bias. In the same line, the proportion of HPs who declared having been trained either during their initial formation or thereafter during a secondary training (16.8% and 14.2% respectively) is also above the results previously reported by others studies in France and in the US [12,13]. In addition, we cannot rule out an interpretation bias due to the way questions were formulated.

Another limitation is the overrepresentation of midwives (41%) versus other medical professionals. The PACA region is one of the largest regions, with the third most important midwife demography in the country and a dynamic natality (57,689 births in 2016). We cannot rule out that our MW population is not fully representative of the national MW population. However, Marie and colleagues [18] recently published a cross-sectional study on the perception of environmental risks by perinatal health professionals in a central region of France. Both studies present consistent results: (1) a minority of health professionals asked pregnant women about their chemical exposure and advised them to reduce these exposures; and (2) lack of information, training and scientific evidence were the barriers invoked to explain this attitude. Taken together, the results of both works argue for a possible extrapolation to the entire country. Both studies illustrate the importance of engaging in action on environmental health to inspire the training of future perinatal HPs concerning this topic and to advocate for interventions based on Developmental Origin of Health and Disease concepts as well as to build new initiatives to promote a healthy start to life. Information should be routinely conveyed in antenatal clinics, including during preconception visits, so that women are made aware of key facts that will allow them to make informed choices regarding their lifestyle.

## 5. Conclusions

Since 2015, several international scientific societies have urged reproductive health professionals, including obstetricians, gynaecologists, midwives, nurses, women’s health nurse practitioners, nursery nurses and all other professionals working in the field of neonatology and early infancy, to include in the clinical setting some actions to prevent exposure to harmful environmental agents. Translating science into easy to understand messages and easy to implement proactive measures is urgent to protect the most vulnerable populations: young children, women and men of child-bearing age and expectant mothers. This urgency requires altogether awareness, adhesion and knowledge from perinatal HPs regarding EH. In this work, we have shown that in southern France, a large panel of perinatal HPs is concerned about the EH matter. HPs have rather good knowledge regarding environmental toxicants but fail to put it into systematic daily practice. Only a few of them have received specific information during their initial training, and only a small subset has followed secondary training. HPs specifically called for training, synthetizing evidence-based medicine, which includes practical guidance for the counselling and transfer of patients. If specific training information programmes exist in France, in the fields of environment, endocrinology and cancer, we need to set up adequate programmes dedicated to perinatology responding to the demand of perinatal HPs. Our study emphasizes the need to set up such secondary training but also, and importantly, to include EH in initial training so that HPs of all backgrounds make EH part of their cultural background.

## Figures and Tables

**Table 1:** Socio-demographic, professional characteristics and geographic area of practice of perinatal health professionals (questionnaire survey).

	n = 962
**Gender n (%)**	843 ^a^
Male	59 (7%)
Female	784 (93%)
**Age—years (mean ± SD)**	822 ^b^
<42 years	400 (48,7%)
>42 years	422 (51.8%)
**Length of service n (%)**	839 ^c^
<1 year	42 (5%)
1 to 5 years	117 (13.9%)
5 to 10 years	153 (18.2%)
>10 years	527 (62.8%)
**Geographic area of practice n (%)**	837 ^d^
Bouches du Rhône (Marseille)	432 (51.9)
Alpes Maritimes (Nice)	131 (15.7)
Var (Toulon)	95 (11.4)
Vaucluse (Avignon)	70 (8.4)
Alpes de Hautes-Provence	35 (4.2)
Hautes-Alpes	26 (3.1)
Monaco Corse	5 (0.6)
Corse	38 (4.6)

^a^, ^b^, ^c^, ^d^ number of participants who gave this information () main city.

**Table 2:** General knowledge in Environmental health (1/3).

	Answer				
Question Expected Responses [3,22,23,24,25,26,27]	Mean ± SD	Correct and Extreme n (%)	Correct and Unsure n (%)	Wrong n (%)	Does not Know n (%)
To prevent viral infection, pregnant women are advised not to air their dwelling place—fully disagree	4.84 ± 0.62	828 (91.2)	45 (5)	22 (2.4)	13 (1.4)
Outdoor pollutants are not harmful for children under 3 as their body is not yet sensitive to it—fully disagree	4.78 ± 0.73	801 (88.2)	64 (7)	32 (3.5)	11 (1.2)
Cigarette smoke spreading in the room persists after the smoker’ exit—strongly agree	4.79 ± 0.65	776 (86.6)	93 (10.4)	20 (2.2)	7 (0.8)
In utero exposure to endocrine disruptors do not impact the foetus when organogenesis is incomplete—fully disagree	4.72 ± 0.83	785 (86.5)	62 (6.2)	40 (4.4)	21 (2.3)
In humans impacts of environmental toxicants can be discovered several years after exposure as they sometimes display delayed effects—strongly agree	4.74 ± 0.56	713 (78.5)	170 (18.7)	9 (1)	16 (1.8)
Pregnant women are advised to burn incense and use air fresheners to relax—fully disagree	4.67 ± 0.67	685 (76.5)	149 (16.6)	15 (1.7)	47 (5.2)
Exposure to pesticide can affect male fertility—strongly agree	4.68 ± 0.64	683 (75.2)	176 (19.4)	9 (1)	40 (4.4)
To avoid exposure to endocrine disruptors it is recommended to use plastics food containers—fully disagree	4.55 ± 0.82	634 (70.8)	155 (17.3)	24 (2.7)	83 (9.3)
Pregnant women can enter in a freshly painted room as soon as paint is dry without exposing themselves to chemicals—fully disagree	4.40 ± 0.89	536 (59.8)	241 (26.9)	43 (4.8)	76 (8.5)
Plastic baby bottles are recommended instead of glass bottles—fully disagree	4.30 ± 1.06	540 (59.5)	221 (24.3)	91 (10.6)	51 (5.6)
Indoor air can be more polluted than outdoor’—strongly agree	4.39 ± 0.93	526 (58.7)	272 (30.4)	56 (6.2)	51 (5.6)
In humans all diseases due to environmental toxicants are reversible—fully disagree	4.32 ± 0.91	493 (54.3)	273 (30.1)	48 (5.3)	94 (10.4)
Pregnant women are advised to use sprayed cosmetics and cleaning agents—fully disagree	4.26 ± 0.92	486 (54.2)	188 (21)	26 (2.9)	196 (21.9)
Release of endocrine disruptors present in plastic food containers is increased by heating process—strongly agree	4.40 ± 0.76	469 (53.8)	300 (34.4)	14 (1.6)	88 (10.1)
Ingestion and inhalation are the sole route of exposure to environmental toxicants—fully disagree	4.08 ± 1.08	406 (44.5)	326 (35.7)	130 (14.3)	50 (5.5)
Pregnant women are advised to prefer fatty fish (salmon, sardines, and mackerel) to lean fish (cod, hake) as heavy metals contained in lean fish are not harmful. Strongly disagree	3.79 ± 1.14	328 (36.6)	195 (21.8)	114 (12.7)	259 (28.9)
pesticides are eliminated by careful rinsing of fruits and vegetables—fully disagree	3.65 ± 1.24	251 (28.0)	366 (40.8)	246 (27.5)	33 (3.7)
Pregnant women are advised to use rinse-off hygiene products (e.g., soap instead of cleaning lotion) for themselves and their child under 3 as they are less skin permeable and less harmful—strongly agree	3.58 ± 0.96	146 (16.8)	332 (38.1)	93 (10.3)	300 (34.4)
All baby products made in France do not contain toxic agents—fully disagree	4.07 ± 0.98	350 (40.2)	329 (37.8)	84 (9.6)	108 (12.4)
To reduce electromagnetic waves exposure, it is advisable to place the baby monitor at least 1m away from the bed—strongly agree	3.65 ± 0.89	137 (15.7)	384 (44.1)	68 (7.8)	282 (32.4)
Environmental risks for male and female fertility are essentially the same—strongly agree	3.17 ± 1.08	87 (10.0)	290 (33.3)	269 (30.9)	225 (25.8)
Mothers are advised to feed fish to children under 3 at least 2 times a week—fully disagree	2.65 ± 1.10	29 (3.3)	185 (21.2)	392 (45.0)	265 (30.4)
Consuming organic food can reduce obstetrical complications—strongly agree	2.81 ± 1.11	46 (5.3)	204 (23.4)	333 (38.2)	288 (33.1)
It is generally advised to wait 2 months before installing baby in his freshly redecorated/refurbished bedroom strongly agree	3.37 ± 0.85	81 (9.0)	287 (32.0)	95 (10.6)	433 (48.3)
Pregnant women are advised to eat fatty fish at least 2 times a week—fully disagree	3.06 ± 1.26	139 (15.3)	240 (26.4)	365 (40.2)	164 (18.1)

**Table 3:** Difficulties cited by HPs in confront of Environmental Health (open-ended question), thematic analysis and numbers.

Themes	n (%)	Qualitative data
Paucity of knowledge	403 (73.7)	Lack of knowledge regarding toxicants and their relevance to the geographic area of practice. Lack of scientific proof, evidence-based medicine data and professional recommendation. Poor knowledge of what it is possible to advice. lack of training
Fear of patient’ reaction	52 (9.5)	Patients seen as poorly concerned by the EH problem, fear of evoking guilt and stress, fear of being seen as a patronizing
Inability to provide solution Lack of solution	63 (11.5)	Expensive solutions, no solution to provide (particularly with respect to occupational exposure), benefice/risk balance difficult to evaluate, lack of support or resource (institutional or colleague)
Pregnancy follow up visit: an inappropriate time	36 (6.6)	Lack of time, lack of tools to explains (leaflets, brochures, posters)
Communications issues	22 (4.0)	Lack of communication skills, differences in risk perception between professionals and patients due to culture or language skills, patient non-fluent in French
Multiple sources of information/wealth of scientific data	16 (2.9)	Large body of literature and information difficult to sort. A scientific field in constant evolution. Lack of time to find pertinent information
Environment/society conflict of interest	11 (2.0)	Conflict of interest between consumerism and health advices, lack of action taken by public authorities and governments bodies. Lobbying of companies
Need of coordination between professionals	4 (0.7)	Need of coordination between professionals
Multiplicity of prevention messages	3 (0.5)	Multiplicity of prevention messages
Not a priority	4 (0.7)	Priority settings (Medical aspects)
Daily habits difficult to change	7 (1.3)	Daily habits difficult to change

**Table 4:** Environmental problems faced by HPs in their area of practice (open-ended question): thematic analysis and figures.

Themes	n (%)	Qualitative Data
Pollution	292 (40.1)	Air pollution, industrial pollution, plants and factories, drinking water pollution, vehicle exhaust fumes, sea pollution, soil pollution
Daily hygiene and cleaning products	147 (20.2)	Cosmetic and hygiene products, hair dyes, baby products, nappies, cleaning products, plastics, baby bottles, bisphenols, plastics toys, sprays, clothes
Inadequate housing	78 (10.7)	Lead, lead poisoning, insalubrious housing, asbestos, substandard housing
Pesticides	99 (13.6)	Pesticides
Occupational exposures	63 (8.6)	Use of toxic cleaning products, poor premises ventilation, hospital wastes, disposable medical devices and consumables, plastic consumables, poor recycling, baby hygiene products and cosmetics, inappropriate use of disinfecting hand products, X-rays, paints, virus and bacterial exposure, nosocomial infections, anesthetic gas, lack of day light, use of smelling products, rehabilitation of hospital premises, hospital food
Endocrine disruptors	62 (8.5)	Endocrine disruptors
Diet	68 (9.3)	Diet during pregnancy and breastfeeding, baby food
Indoor air	38 (5.2)	Indoor air quality, Carbon monoxide intoxication, heating system, ventilation, air conditioning, furniture, paints, refurbishing, solvents
Toxic consumption	35 (4.8)	Tobacco smoking, alcohol, drugs, addictions, medications
Electromagnetic waves	25 (3.4)	Micro waves, Wi-Fi, electromagnetic waves
Wastes	23 (3.2)	Wastes, absence sorting of recycling
Socio-economic level	30 (4.1)	Precariousness, economic costs, socio-economic difficulties
Noise	8 (1.1)	Noise

## Supplementary Materials

The following are available online at http://www.mdpi.com/1660-4601/15/10/2259/s1, Table S1: Structure of the questionnaire, Table S2: Size and list of occupation represented in the questionnaire survey, Table S3: Representation of Environmental health: content thematic analysis.
