# Supplementary material for: Environmental Health in Perinatal and Early Childhood: Awareness, Representation, Knowledge and Practice of Southern France Perinatal Health Professionals
Source: Int J Environ Res Public Health. 2018 Oct 15;15(10):2259. doi: 10.3390/ijerph15102259 (PMC6211056; doi:10.3390/ijerph15102259)
Supplement: Supplementary file 1 [file ijerph-15-02259-s001.pdf]

**Supplementary Materials**

**Table S1**: Structure of the questionnaire

| | | | | | |
|---|---|---|---|---|---|
| 3 words or expressions arising to you when you read 3 Environmental health<br>(Followed by WHO 's definition of Environmental Health)<br>1.<br>2.<br>3. | | | | | |
| **Q1 to Q25**: See table 2 of the manuscript | 1 = fully disagree | 2 = partially disagree | 3 = does not know | 4 = rather agree | 5 = strongly agree |
| **Q 26:** According to you among these propositions, which one(s) are potential source of lead exposure? (multiple choice question) | | | | | |
| Some traditional make-up (Kohl eye-liner) | Tap water | Own grown fruits and vegetables | Some paints | Some toys | |
| **Q 27:** According to you among these propositions, which one(s) are potential source of exposure to endocrine disruptors (multiple choice question) | | | | | |
| Natural cleaning products (Black soap, Marseille's soap | Some toys | Some cosmetic and hygiene products | Food glass containers | | |
| **Q 28:** (Open question) According to you what are the dominant environmental issues in you geographic area of practice | | | | | |
| **Q 29:** In your professional daily practice, do you feel concerned by environmental issues? | | | | | |
| 1 = not | 2 = not really | 3 = mildly | 4 = rather | 5 = fully concerned | |
| **Q 30:** Do you feel the necessity to inform your patients regarding environmental health issues? | | | | | |
| 1 = not | 2 = not really | 3 = mildly | 4 = rather | 5 = fully concerned | |
| **Q 31:** Do you face difficulties to inform your patients regarding environmental health issues | | | | | |
| 1 = never | 2 = seldom | 3 = sometime | 4 = often | 5 = always | |
| **Q 32:** (Open question) According to you what are the reasons you are facing difficulties to inform patients? | | | | | |
| **Q 33:** Are you facing difficulties to refer patients to experts? | | | | | |
| 1 = never | 2 = seldom | 3 = sometime | 4 = often | 5 = always | |
| **Q 34:** How would you evaluate your knowledge in Environmental health? | | | | | |
| 1 = null | 2 = poor | 3 = average | 4 = good | 5 = excellent | |
| **Q 35:** Would you be interested in a professional training in Environmental Health in the field of perinatalogy and fertility | | | | | |
| 1 = not | 2 = not really | 3 = mildly | 4 = rather | 5 = fully interested | |
| Socio-demographic questions<br>Age<br>Sex<br>Professional postal code<br>Profession<br>Year of qualification<br>Length of service<br>Environmental Health initial training<br>Environmental Health secondary training<br>Mean of information on the subject | If YES which one? | | | | |

**Table S2:** Size and list of occupation represented in the questionnaire survey

| Occupation | n= 843 |
|---|---|
| Midwife | 346 |
| Nursery nurse | 93 |
| Pediatrician | 74 |
| Obstetrician | 74 |
| General Practitioner | 46 |
| Nurse | 42 |
| Nursery nurse assistant | 34 |
| Social worker | 21 |
| Gynecologist | 21 |
| Family home visitor | 21 |
| Nursery director | 17 |
| Nutritionist | 12 |
| Psychologist | 13 |
| Health care manager | 3 |
| Endocrinologist | 3 |
| Medical secretary | 2 |
| Speech therapist | 2 |
| Nursing assistant | 2 |
| Pediatric psychiatrist | 3 |
| Chaplain | 2 |
| Teacher | 2 |
| Anesthesiologist | 2 |
| Physiotherapist | 2 |
| Administrative assistant | 1 |
| Radiologist | 1 |
| Geneticist | 1 |
| Housing health advisor | 1 |
| Biologist | 1 |

**Table S3: Representation of Environmental health: content thematic analysis** (1/6)

| Theme | Number of response Frequency n (%) [a] | Sub-theme | Frequency n (%) [b] | Qualitative data |
|---|---|---|---|---|
| **Theme 1**: Toxic agents and environmental health risks | 613 (68) | Pollution | 553 (73.9) | Pollution(s), pollutants, impact of pollution, air quality pollution, air quality, atmospheric pollution, fine particles, water pollution, water quality, Earth |
| | | Toxicants in food | 262 (42.7) | Pesticides, food quality, food products, nutrition, healthy food, food contamination, unhealthy diet, food additives, organic, local agriculture, genetically modified micro-organisms |
| | | Endocrine Disruptors | 120 (19.6) | Endocrine disruptors, disrupting agents, endocrine inducers, hormonal disruptors |
| | | Physical and chemical agents | 36 (6.4) | Chemistry, chemicals, bisphenol, parabens, asbestos, phthalate, heavy metals, lead, gasoil, perfumes, solvents, paints and varnishes, medication |
| | | Addiction | 21 (3.4) | Tobacco, smocking, passive smoking, drugs and toxics |
| | | Noise | 15 (2.4) | Noise, noise nuisance, noise pollution |
| | | Household and Everyday objects | 14 (2.3) | Plastics, daily products, furniture, aerosol |
| | | Waves | 13 (2.1) | Waves, Wi-Fi, micro-waves |
| | | Stress | 10 (1.6) | Stress, work related stress |
| | | Cosmetics and hygiene products | 9 (1.5) | Hygiene products, cosmetics, beauty products, baby care products |
| | | Waste | 7 (1.1) | Wastes |
| | | House cleaning products | 6 (1) | Cleaning agents, detersives, household products |
| | | Light | 3 (0.5) | Light, light pollution |

[a] with respect to the number of HPs participant to the survey, [b] with respect to the number of HPs who cited this sub-theme

**Table S3: Representation of Environmental health: content thematic analysis** (2/6)

| Theme | Number of response Frequency n (%) [a] | Sub-theme | Frequency n (%) [b] | Qualitative data |
|---|---|---|---|---|
| **Theme 2**: Collective means of action (society and communities) | 293 (32.5) | Act for earth protection and respect for the environment | 163 (55.6) | Protection, protect, environmental compliance, respect, respect for the environment, welfare, caring, ecology, green, sustainable development, renewable energy, energy saving, waste recycling, zero waste, |
| | | Develop prevention, inform and raise awareness of the public | 131 (44.7) | Prevention, anticipation, information, raise awareness, vaccination campaign, screening, health promotion, health education, therapeutic patient education |
| | | Patient care proposition | 19 (6.5) | Care, care project, care pathway, care, listening, support |
| | | Young age education | 12 (4.1) | Education, school |
| | | Promotion of specialized health care establishment | 11 (3.8) | Hospitals, Mother Infant Care Centres, medico-social centres, health care centres, medical facilities, medical home visit |
| | | Coordinate and facilitate health professionals collaboration | 7 (2.4) | Social and medical networks, multidisciplinary care, team working, inter-professionals collaboration |
| | | Organization of training courses | 3 (1) | Training, conferences, scientific meetings |
| | | Promote medical research | 2 (0.7) | Research |
| | | Care management recommendation writing | 1 (0.3) | Recommendation for health professionals |

[a] with respect to the number of HPs participants to the survey, [b] with respect to the number of HPs who cited this sub-theme

**Table S3: Representation of Environmental health: content thematic analysis** (3/6)

| Theme | Number of response Frequency n (%) [a] | Sub-theme | Frequency n (%) [b] | Qualitative data |
|---|---|---|---|---|
| **Theme 3**: Individual means of action | 73 (8.1) | Healthy way of life | 35 (47.9) | Menstrual cups, reusable nappies, environmental friendly products, healthy and safe products, chose natural materials, modification of comportments, good practices, consume differently |
| | | Physical activity | 22 (15.1) | Physical activity, sport, physical exercise promotion |
| | | Precautionary principle | 6 (8.2) | Precautionary principle, caution |
| | | Ventilate premises | 4 (5.5) | Ventilate, provide better indoor quality |
| **Theme 4**: Important issue, for today and for the future | 94 (10.4) | A long-term issue | 34 (36.2) | Future, future generation, issue for the future, an absolute necessity for human being, In the long run |
| | | A big issue | 30 (31.9) | Important, essential, key focus, vital, compulsory, emergency, risk, warning, to by studied, disaster |
| | | Environment perceived as a health threat | 21 (22.3) | Epidemic, disease outbreak, contamination, contagion, infection, viruses, parasites, poison, poisoning, intoxication |
| | | Current issue | 9 (9.6) | Current issue, topical subject, fashionable issue |
| | | challenge | 8 (8.5) | Powerlessness, a lot to be done, hard, challenging, lack of means of action, fight |
| **Theme 5**: adverse consequences of the environment on human health | 89 (9.9) | Pathologies | 32 (36) | Pathologies, diseases, syndrome, obesity |
| | | Cancers | 28 (31.5) | Cancers |
| | | Allergies | 24 (27) | Allergies, allergens |
| | | Reprotoxicity | 22 (4.7) | Reprotoxic, reprotoxicity, fertility, infertility, reproductive health, preconception |

[a] with respect to the number of HPs participant to the survey, [b] with respect to the number of HPs who cited this sub-theme

**Table S3: Representation of Environmental health: content thematic analysis** (4/6)

| Theme | Number of response Frequency n (%) [a] | Sub-theme | Frequency n (%) [b] | Qualitative data |
|---|---|---|---|---|
| **Theme 6**: Environmental health more than absence of disease | 164 (18.2) | Wellbeing | 87 (53) | Wellbeing |
| | | Quality of life | 44 (26.8) | Quality of life |
| | | Better life | 21 (12.8) | Everyday comfort, to live better, harmony, good balance, to be in good shape, living healthy and happy, pleasure, serenity, beauty, relaxation |
| | | Social support | 16 (9.8) | Acquaintance, social support |
| | | Feeling secure | 14 (8.5) | safety |
| **Theme 7**: Impact of socio-demographic factors in environmental health | 101 (11.2) | Living condition | 46 (45.5) | Daily life, life circumstances, everyday lives, lifestyle, way of life, everyday comfort, daily habits, living space, living environment |
| | | Housing | 27 (26.7) | Accommodation, housing, house, home |
| | | Socio-economic disparities | 22 (21.8) | Socio-economic inequality, social background, precariousness, poverty, social circumstances |
| | | Working environment | 12 (11.9) | Working environment, workplace, professional dimension |
| | | Access to the care system | 2 (2) | Access to healthcare |
| **Theme 8**: Collective responsibility | 56 (6.2) | A public health issue | 24 (42.9) | Public health |
| | | A responsible and collective issue | 20 (35.7) | Everyone's responsibility, responsible health concept, collective awareness, solidarity, altruism, collective action, civism |
| | | All concerned | 15 (26.8) | All concerned, general interest, universality, everybody impacted, general public |
| **Theme 9**: Places at risk of toxic exposure | 44 (4.9) | Houses | 27 (61.4) | Houses, accommodation, home |
| | | Working place | 12 (27.3) | Working place, working environment |
| | | Urban and industrial areas | 5 (11.4) | Industry, toxic exposures from the industry, cities |

**Table S3: Representation of Environmental health: content thematic analysis** (5/6)

| Theme | Number of response Frequency n (%) [a] | Sub-theme | Frequency n (%) [b] | Qualitative data |
|---|---|---|---|---|
| **Theme 10**: perinatal health a gateway to develop environmental health | 12 (1.3) | perinatal health a gateway to develop environmental health | 12 (100) | Epigenetics, pregnancy, fetal impregnation, breastfeeding |
| **Theme 11**: definition of environmental health | 166 (18.4) | environmental health and general health relation | 64 (38.6) | Health, good health, "A healthy spirit in a healthy body", healthy, healthy environment |
| | | Nature and health relation | 54 (32.5) | Nature, forest, plants, trees, mountain, country side, sun, see side, world, Earth |
| | | Impact of environment on health | 30 (18.2) | Impact (or consequences) of environment on health |
| | | A large field | 8 (4.8) | Global, huge |
| | | Human adaptation to the environment | 8 (4.8) | Adaptation to the environment, ergonomy, adaptation to the evolution of the society, conflictual relationship with the evolving society |
| | | Environment: everything around us | 5 (3) | Environment: everything around us |
| | | Health and environment not dissociable | 4 (2.4) | Environment dependency, goes hand in hand |
| | | No definition | 4 (2.4) | What is it? No idea, it's nothing |

[a] with respect to the number of HPs participant to the survey, [b] with respect to the number of HPs who cited this sub-theme

**Table S3: Representation of Environmental health: content thematic analysis** (6/6)

| Theme | Number of response Frequency n (%) [a] | Sub-theme | Frequency n (%) [b] | Qualitative data |
|---|---|---|---|---|
| **Theme 12**: Move toward action for environmental health | 23 (2.5) | Under estimated issue | 10 (43.5) | inadequately assessed, not done enough in term of scientific research, social denial, inaction, not enough prevention, unawareness, not taken into account (in health care), a problem marginalized, lack of knowledge |
| | | Toward an improvement | 7 (30.4) | Evolution, how to improve, reflection, progress, a new paradigm |
| | | Raise awareness | 3 (13) | Awareness |
| | | commitment | 3 (13) | Investment, commitment |
| **Theme 13**: other political issues in environmental health | 24 (2.7) | Economical and commercial issues | 8 (33.3) | Economy, economic issues, economic interests, financial profits, commercial interests, health economy, health costs |
| | | Political and economic choices | 6 (25) | Politics, political issues, public authorities, stakeholders |
| | | Public authorities transparency | 6 (25) | Public authorities ignorance, politically correct, hypocrisy, transparency, honesty, hoax |
| | | Lobbying | 5 (20.8) | Lobbying, influence, industrial lobbies |
| **Theme 14**: Mode of exposure | 12 (1.3) | Inhalation | 11 (91.7) | Breath, breathing, inhalation |
| | | Skin contact | 1 (8.3) | Contact with the skin |
| **Theme 15**: Environmental damage | 14 (1.6) | Environmental degradation | 14 (100) | Mundialization, climate change, urbanism, deforestation |
| **Theme 16**: Anthropocentric appreciation | 39 (4.3) | A human issue Centered on mankind | 39 (100) | Human, mankind, children, newborns, family, parents, population(s) |
| **Theme 17**: Refer to WHO | 4 (0.4) | Refer to WHO | 4 (100) | WHO |

[a] with respect to the number of HPs participant to the survey, [b] with respect to the number of HPs who cited this sub-theme
